# Multi-capillary column-ion mobility spectrometry (MCC-IMS) as a new method for the quantification of occupational exposure to sevoflurane in anaesthesia workplaces: an observational feasibility study

**DOI:** 10.1186/s12995-015-0056-7

**Published:** 2015-03-27

**Authors:** Nils Kunze, Cathrin Weigel, Wolfgang Vautz, Katrin Schwerdtfeger, Melanie Jünger, Michael Quintel, Thorsten Perl

**Affiliations:** Department for Anaesthesiology, Centre for Anaesthesiology, Emergency and Intensive Care Medicine, University Medical Centre, University of Göttingen, Robert-Koch-Straße 40, 37075 Göttingen, Germany; Leibniz-Insitut für Analytische Wissenschaften – ISAS – e. V, Bunsen-Kirchhoff-Straße 11, 44139 Dortmund, Germany; Department of Molecular Plant Genetics, University of Hamburg, Ohnhorststraße 18, 22609 Hamburg, Germany

**Keywords:** Sevoflurane, Occupational exposure, Volatile anaesthetics, Ion mobility spectrometry, Room air analyses, Limit of detection

## Abstract

**Background:**

Occupational exposure to sevoflurane has the potential to cause health damage in hospital personnel. Workplace contamination with the substance mostly is assessed by using photoacoustic infrared spectrometry with detection limits of 10 ppbv. Multi-capillary column-ion mobility spectrometry (MCC-IMS) could be an alternative technology for the quantification of sevoflurane in the room air and could be even more accurate because of potentially lower detection limits. The aim of this study was to test the hypothesis that MCC-IMS is able to detect and monitor very low concentrations of sevoflurane (<10 ppbv) and to evaluate the exposure of hospital personnel to sevoflurane during paediatric anaesthesia and in the post anaesthesia care unit (PACU).

**Methods:**

A MCC-IMS device was calibrated to several concentrations of sevoflurane and limits of detection (LOD) and quantification (LOQ) were calculated. Sevoflurane exposure of hospital personnel was measured at two anaesthesia workplaces and time-weighted average (TWA) values were calculated.

**Results:**

The LOD was 0.0068 ppbv and the LOQ was 0.0189 ppbv. During paediatric anaesthesia the mean sevoflurane concentration was 46.9 ppbv (8.0 - 314.7 ppbv) with TWA values between 5.8 and 45.7 ppbv. In the PACU the mean sevoflurane concentration was 27.9 ppbv (8.0 – 170.2 ppbv) and TWA values reached from 8.3 to 45.1 ppbv.

**Conclusions:**

MCC-IMS shows a significantly lower LOD and LOQ than comparable methods. It is a reliable technology for monitoring sevoflurane concentrations at anaesthesia workplaces and has a particular strength in quantifying low-level contaminations of sevoflurane. The exposure of the personnel working in these areas did not exceed recommended limits and therefore adverse health effects are unlikely.

## Background

Chronic occupational exposure to the volatile anaesthetic sevoflurane has been discussed to have negative influence on the health of hospital personnel since it was introduced into clinical use in the 1990’s [1,2]. However, evidence on the adverse effects of low-level exposure to volatile anaesthetics stays inconclusive [3]. Governmental organisations of most countries defined occupational exposure limits (OEL) for the first generation of volatile anaesthetics (N_2_O, Halothane). To our knowledge only the Netherlands with 18,700 parts per billion by volume (ppbv) defined an OEL for sevoflurane, by means of a time-weighted average over an 8-hour working day (TWA-S) [4]. For the whole group of halogenated inhaled anaesthetics different countries recommended different TWA-S values between 2,000 and 50,000 ppbv [5,6]. Regarding the potential health damage caused by chronic occupational exposure to sevoflurane the National Institute for Occupational Safety and Health (NIOSH) of the United States recommended a ceiling value of 2,000 ppbv for health care workers [7].

The contamination of workplaces by sevoflurane has been assessed previously. Most authors used photoacoustic infrared spectrometry (PIS) and lower detection limits were indicated in a range between 9 ppbv and 20 ppbv [4,8-11]. Other authors used proton transfer reaction-mass spectrometry (PTR-MS) for measuring sevoflurane [12-14]. When coupled with a time-of-flight analysis (PTR-ToF-MS), the limits of detection (LOD) and quantification (LOQ) of the method were indicated as 19.86 ppbv and 27.58 ppbv [13].

With this present study we would like to introduce multi-capillary column-ion mobility spectrometry (MCC-IMS) as a fast and reliable method for monitoring the occupational exposure of hospital personnel to sevoflurane. The technology of ion mobility spectrometry (IMS) is widely used by security services and by the military for the detection of illegal substances and explosives [15]. Coupling the IMS device with a multi-capillary column for pre-separation allows the analysis of complex and humid gas samples with substance specific detection limits down to parts per trillion (ppt). MCC-IMS technology has been used for a multitude of analytical questions in a medical surrounding, including breath gas analyses for diagnostic purposes, pharmacological monitoring or in-vitro analyses for the differentiation of human pathogenic microbes [16-20]. MCC-IMS provides direct and very fast detection (within seconds) and is able to detect more than one substance at once.

With our present study we want to answer the question if MCC-IMS is a suitable analytical tool for the quantification of workplace concentrations of sevoflurane. Therefore, we calibrated a MCC-IMS device to several sevoflurane concentrations and calculated the LOD and the LOQ of the method. To give an example of the performance of the MCC-IMS in quantifying sevoflurane and to answer the question if there is a relevant exposure to sevoflurane in our institution, we performed room air measurements during paediatric anaesthesia and in the PACU of our University Medical Centre. Both anaesthesia workplaces are at risk for higher sevoflurane exposures, by means of sevoflurane concentrations and TWA (−S) values above international recommendations [13,14,21]. Therefore these areas seemed of particular interest for occupational health research and were chosen for our measurements.

## Methods

According to the regulations of the University Medical Centre of Göttingen no approval from the ethical committee was needed, as this study did not include any of our patient’s data or any medical interventions. However, the ethical committee of the Faculty of Medicine of the University of Göttingen has been informed about the study protocol.

**Figure 1:**
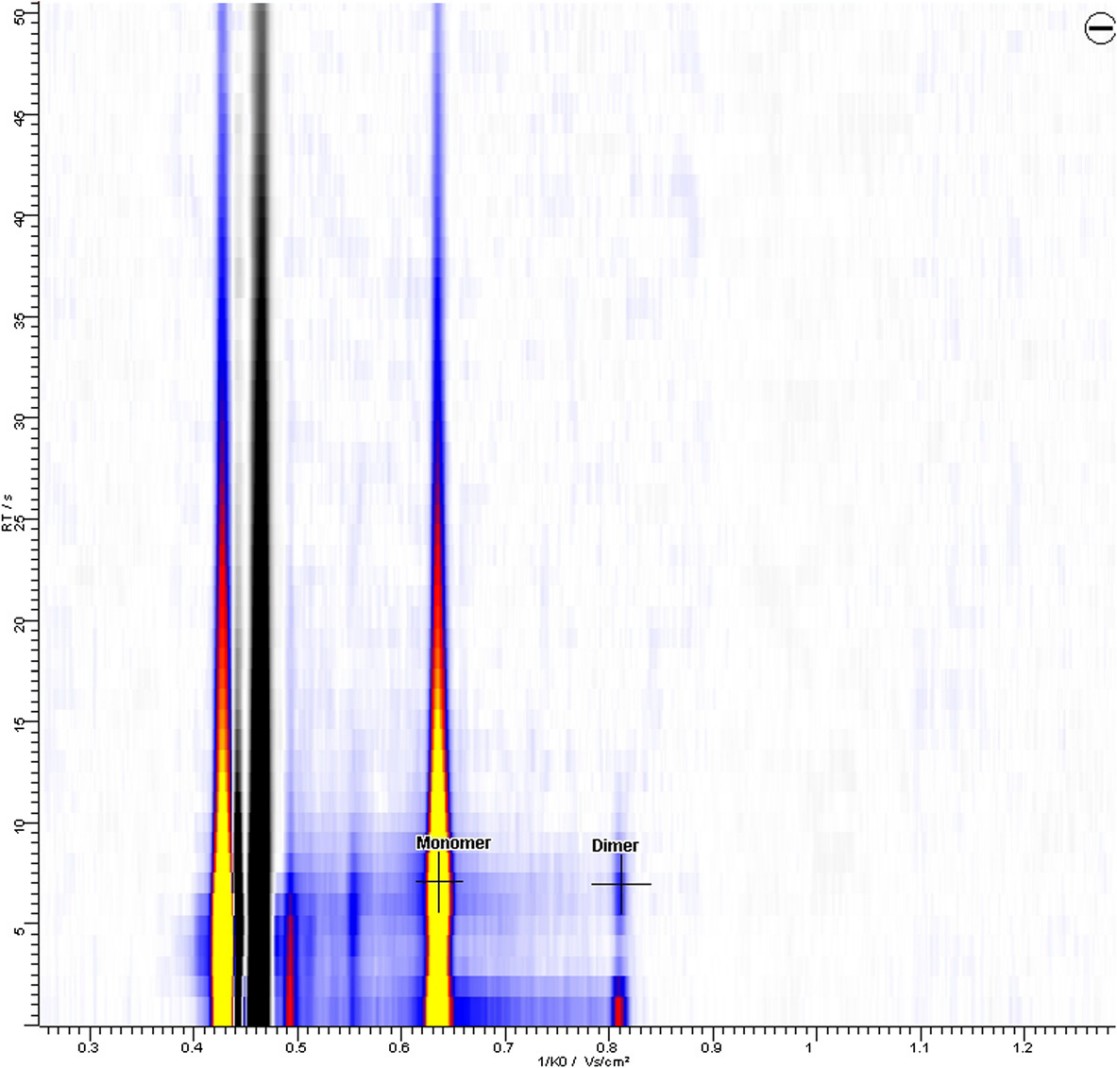
**Topographic MCC-IMS plot marking the positions and intensities of the monomer and the dimer of sevoflurane at a concentration of 200 ppbv during calibration with the x-axis indicating the inverse ion mobility involt seconds per square centimeter (Vs/cm** ^**2**^ **) and the y-axis indicating the MCC retention time in seconds (s).** Signal intensities are indicated by the peak colour, whereas white indicates lowest and yellow highest signal intensities.

Room air measurements were performed during paediatric anaesthesia and in the PACU of the University Medical Centre using a MCC-IMS device (Leibniz-Institut für Analytische Wissenschaften – ISAS – e.V., Dortmund, Germany). The fundamentals of ion mobility spectrometry are described in detail in literature, as well as the principle of gas sample analyses by the MCC-IMS technology has been described in detail previously [15]. We therefore only give a short overview on its principle (see Table 1 for the specifications of the MCC-IMS used for this study): Gas samples are drawn directly by the MCC-IMS’s membrane pump and through an 8 ml sample loop consisting of Teflon. A six-port valve (ISAS, Dortmund, Germany) enables its introduction into the multi-capillary column. After pre-separation, the gas samples are lead into the ionization chamber, where they are ionized through charge transfer from prior ionized reactant ions. A shutter grid that opens for defined range of time (300 μs) separates drift region and ionization region. Ionized molecules pass the shutter grid and get accelerated in a weak electric field towards the detector (Faraday plate) along the drift region of the MCC-IMS. On their way to the detector, ions are separated through collision with drift gas molecules moving in the opposite direction, with their individual velocities depending on size, shape and charge of the molecules. Drift times are measured and the respective drift velocities are calculated for the known drift distances. Ion mobility is calculated by normalizing the drift velocities to the known electrical field. In a last step the reduced ion mobility, which is characteristic to an ion and independent on the experimental conditions, is calculated by normalization to temperature and pressure [22]. In the drift region ions are moving through an external electric field with positive or negative polarity. We used the mode for the detection of the negative ions of sevoflurane. Analyses of the detected MCC-IMS signals were performed using the BB_IMSAnalyse software (Version 1.0, ISAS, Dortmund, Germany). With the MCC working at a temperature of 40°C the peak position of the sevoflurane monomer was found to be 1/K_0_ = 0.635 Vs/cm^2^ at 7 s retention time (see Figure 1). Descriptive statistical analyses and statistical plots were done using statistical software (Statistica 10, StatSoft Inc., Tulsa, USA).

**Table 1:** **Specifications of the MCC-IMS used for the study**

**MCC-IMS device**	**Non-commercial, ISAS, Dortmund, Germany**
Preseparation	Multi-capillary column OV-5 (length 20 cm), Multichrom Ltd., Novosibirsk, Russia
MCC temperature	40°C
Sample loop	Teflon, volume 8 ml
Ionization source	ß-radiation, ^63^Ni
Electric field strength	330 V/cm
Shutter opening time	300 μs
Drift distance	12 cm
Drift and carrier gas	Synthetic air, Air Liquide AG, Düsseldorf, Germany
Drift gas flow	100 ml/min
Carrier gas flow	150 ml/min
Temperature	ambient
Pressure	ambient

**Figure 2:**
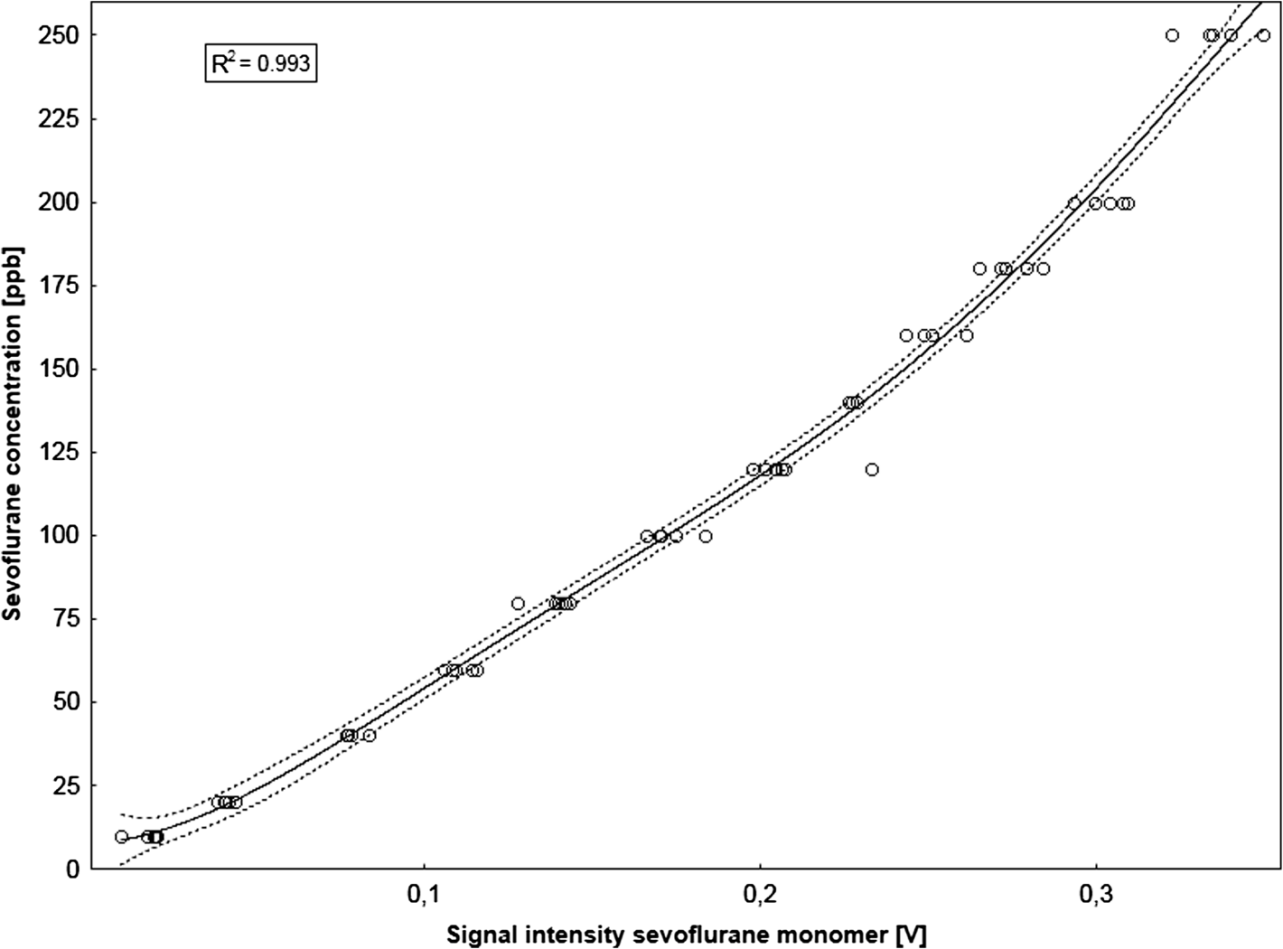
**Results of the calibration of the MCC-IMS to sevoflurane concentration of 10–250 ppbv with the graph indicating the 5th order polynomial correlation of the measurements with a coefficient determination index r** ^**2**^ **= 0.993.** The solid line indicates the regression line and the dotted lines indicate the 95% confidence intervals.

The MCC-IMS was calibrated to a range of sevoflurane concentrations by using a calibration gas generator (HovaCal 3834SC VOC, Inspire Analytical Systems GmbH, Frankfurt am Main, Germany) [23]. Calibration was performed in 40% of relative humidity and with concentrations of 10 – 250 ppbv to simulate common room air humidity and the peak intensity of the sevoflurane monomer peak was correlated to each respective sevoflurane concentration. A 5th order polynomial correlation was found for the results of the calibration measurements, with a coefficient determination index r^2^ = 0.993 (see Figure 2). LOD and LOQ were calculated as described before [16,24].

Room air measurements were performed during 12 paediatric anaesthesia procedures for minor dental surgery during five measurement days between June 19th 2012 and August 7th 2012. All children had general anaesthesia. After mask induction with sevoflurane an intravenous cannula was placed and propofol (B. Braun AG, Melsungen, Germany) was used for the deepening of anaesthesia. Children were intubated using cuffed endotracheal tubes and anaesthesia was maintained by continuous intravenous application of propofol and remifentanil (Ultiva®, GlaxoSmithKline GmbH & Co. KG, Munich, Germany). A commercially available anaesthesia machine with a semi-closed breathing circuit (Cato® edition, Dräger AG, Lübeck, Germany) was used in this study. The anaesthesia machine was connected to the anaesthesia waste gas scavenging system with an air flow of 40 l/min.

Air samples were taken via a 1/8′′ PTFE suction line of 2 m length (Bohlender GmbH, Grünsfeld, Germany) that was attached to the anaesthesia machine in the height of the eyes of the anaesthetist. The first MCC-IMS measurement was started at the beginning of each mask induction. Sampling duration was 30 sec and samples were drawn with 350 ml/min. As sevoflurane was detected at only 7 sec retention time, we performed measurements of 200 s. During the remaining time of the surgical procedure every 13 – 14 minutes another room air measurement was started (up to 70 minutes procedure time). Each measurement was followed by one blank measurement of humid synthetic air in scientific quality (Air Liquid AG, Düsseldorf, Germany) for cleaning the sample loop of the IMS and for controlling the IMS for contaminations. All measurements were performed in two identically constructed operating theatres with room volumes of 95 m^3^ each and air conditioning systems producing air change rates of 3,600 m^3^ per hour.

In addition to our measurements during paediatric anaesthesia we performed room air measurements in the PACU. This central recovery area for all anaesthesia workplaces in our university hospital has a room volume of 715 m^3^ and can take up to 20 patients at once. Air condition is working 24 hours producing air change rates of 3,600 m^3^ per hour. The MCC-IMS was placed in the centre of the PACU and one measurement was performed each hour from October 27th 2009, 10 am until October 29th 2009, 4 pm. Again, each measurement was followed by one blank measurement of humid synthetic air.

According to the recommendations of the German Society for Anaesthesiology and Intensive Care Medicine exposure values were calculated as TWA (time-weighted average) and TWA-S (time-weighted average shift) values. Hereby TWA values were calculated by using the following formula:

1 $$ TWA=\frac{c_1*{t}_1+{c}_2*{t}_2+\cdot \cdot \cdot +{c}_n*{t}_n}{t_1+{t}_2+\cdot \cdot \cdot +{t}_n} $$

Whereas TWA is the time-weighted average (ppbv), c_1_ … c_n_ is the calculated sevoflurane concentration (ppbv) for the measurement 1 to n according to the calibration of the MCC-IMS device, t_1_ … t_n_ is the exposure time (min). To assess the exposure during an average 8-hour working day, we calculated TWA-S values by using the following formula:

2 $$ TWA-S=\frac{TWA*{t}_{\exp }}{8h} $$

Whereas TWA-S is the time-weighted average during an 8-hour shift (ppbv) and t_exp_ is the actual exposure time during the working day of 8 hours.

## Results

### LOD and LOQ

For sevoflurane the lower LOD of the MCC-IMS device used for this study was 0.0068 ppbv and the lower LOQ was 0.0189 ppbv, calculated as described before [16,24]. The MCC-IMS was calibrated using a range of sevoflurane concentrations of 10 – 250 ppbv. On behalf of this setting we were able to describe the lower detection limit and a 5th order polynomial correlation (see Figure 2). With these methods were not able to describe or calculate an upper detection limit. Table 2 shows the LOD and LOQ values of the MCC-IMS in comparison to other analytical methods. Table 3 shows the precision of the calibration.

**Table 2:** **Comparison of LOD and LOQ of the different analytical methods used for the quantification of sevoflurane in the room air**

**Method**	**LOD**	**LOQ**
PIS [4,8-11]	9 – 20 ppbv	-
PTR-MS [12,14]	-*	-
PTR-ToF-MS [13]	19.86 ppbv	27.58 ppbv
MCC-IMS [this study]	0.0068 ppbv	0.0189 ppbv

*No data available; described as “a few parts per trillion”.

**Table 3:** **Results of the sevoflurane calibration: mean signal intensities (SI), standard deviations (SD) and relative standard deviation (RSD%) for the measured sevoflurane concentrations**

**Sevoflurane concentration [ppbv]**	**Mean SI [V]**	**SD [V]**	**RSD [%]**
10	0.0187	0.00535	28.6
20	0.0353	0.00342	9.7
40	0.0638	0.00309	4.8
60	0.0902	0.00294	3.3
80	0.1146	0.00380	3.3
100	0.1438	0.00225	1.6
120	0.1691	0.00573	3.4
140	0.1887	0.00646	3.4
160	0.2107	0.00699	3.3
180	0.2365	0.00543	2.3
200	0.2603	0.00635	2.4
250	0.2944	0.01032	3.5

### Paediatric anaesthesia

**Figure 3:**
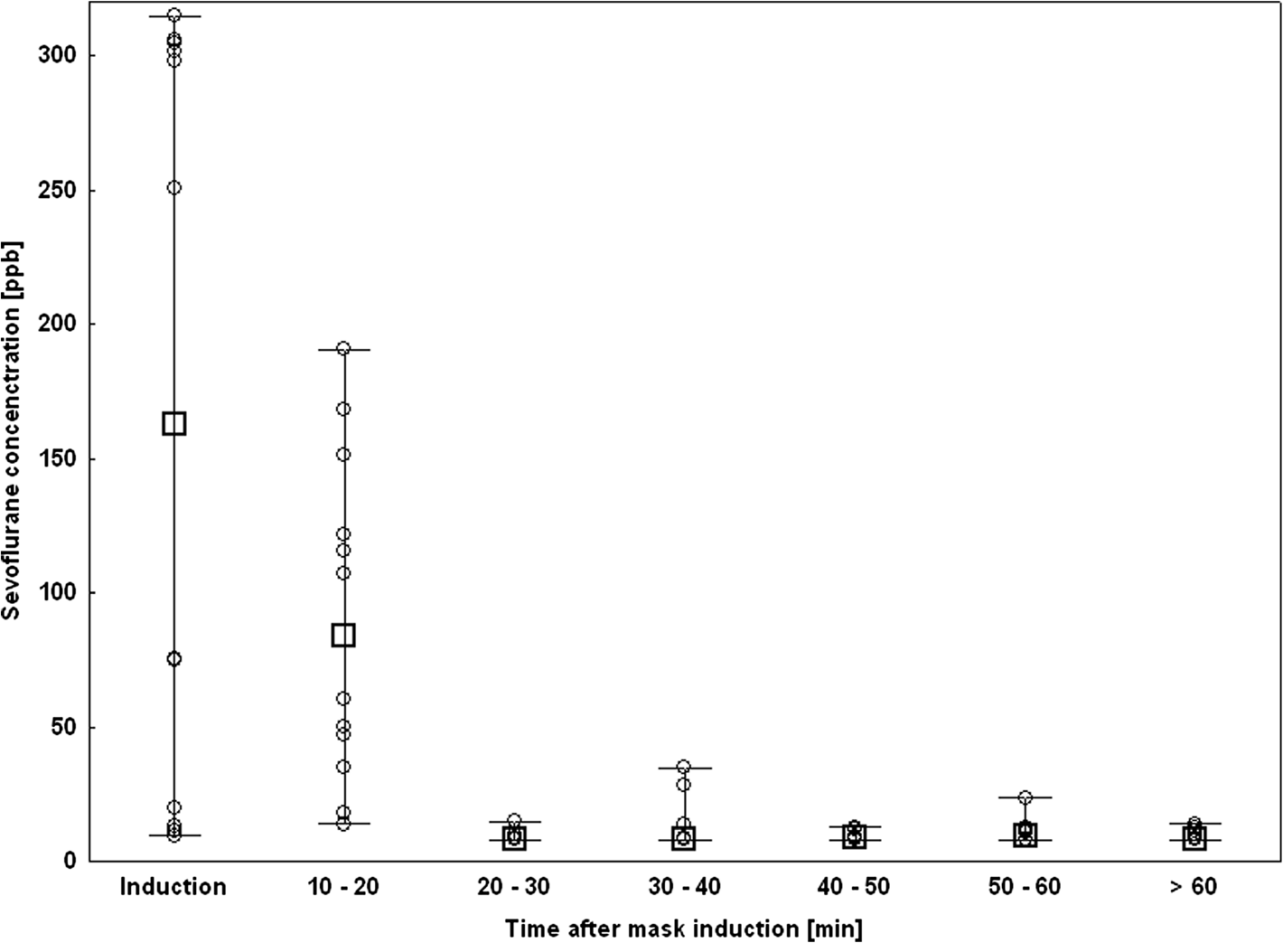
**Results of the room air measurements in the paediatric dental surgical theatre with the whiskers indicating the minimum and maximum concentrations, (**☐**) indicating the median concentrations, and (**○**) indicating single measurement data points.** General anaesthesia was induced by sevoflurane mask induction and maintained as total intravenous anaesthesia.

Room air measurements were performed during paediatric anaesthesia for minor dental surgery in 12 children. The first room air sample was taken at the beginning of the mask induction and during the remaining time of surgery another room air measurement was started every 13 – 14 minutes. In total 77 measurements were performed during the 12 surgical procedures in general anaesthesia. Sevoflurane concentrations reached highest levels in the first measurements (during mask induction) with a minimum of 9.6 ppbv and a maximum of 314.7 ppbv (median value 162.9 ppbv, n = 12). During the on-going time of surgery sevoflurane concentrations were found to decrease, with median values of 83.7 ppbv after 10 – 20 minutes (n = 12), 8.2 ppbv after 20 – 30 minutes (n = 11), 8.2 ppbv after 30 – 40 minutes (n = 11), 8.5 ppbv after 40 – 50 minutes (n = 11), 9.6 ppbv after 50 – 60 minutes (n = 10) and 8.4 ppbv after more than 60 minutes (n = 10). The initial decrease was followed by a phase with almost constantly low concentrations (see Figure 3).

Calculations of the personnel’s actual exposure to sevoflurane during their working days showed TWA values between 22.6 ppbv (July 3rd 2012, Day 2) and 72.9 ppbv (July 17th 2012, Day 4). In relation to an 8-hour working day TWA-S values between 5.8 ppbv (June 19th 2012, Day 1) and 45.7 ppbv (July 17th 2012, Day 4) were calculated from the results of the MCC-IMS measurements. The TWA and TWA-S values are summarized in Table 4.

**Table 4:** **TWA and TWA-S values for hospital personnel working in the paediatric dental surgical theatre**

**Date**	**No. of mask inductions**	**TWA [ppbv]**	**TWA-S [ppbv] (8 hours)**
June 19th 2012	1	37.3	5.8
July 3rd 2012	3	22.6	10.3
July 10th 2012	2	25.4	7.8
July 17th 2012	5	72.9	45.7
August 7th 2012	1	60.8	9.0

### PACU

**Figure 4:**
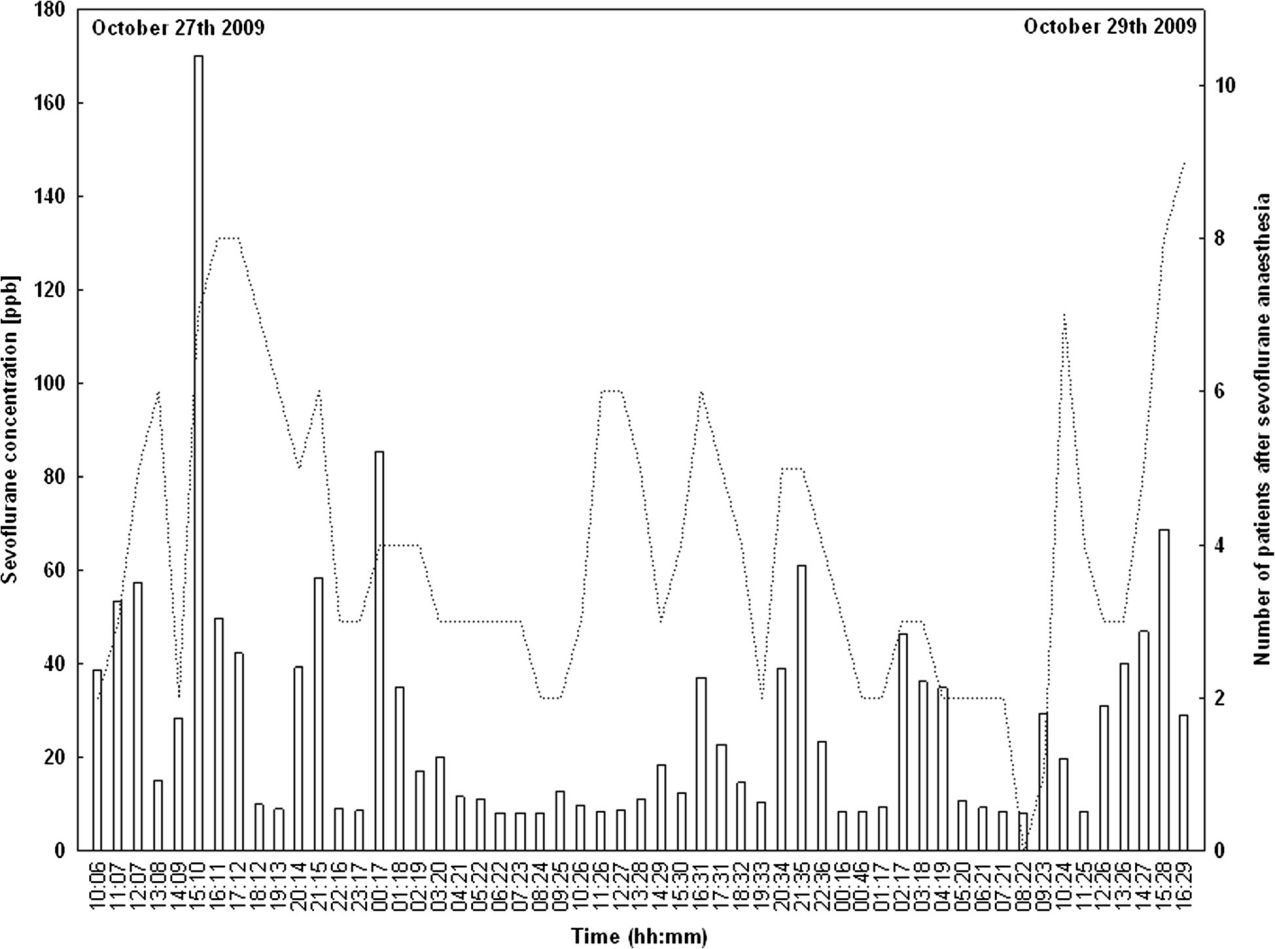
**Results of the room air measurements in the post anaesthesia care unit (PACU).** Measurements were performed between October 27th 2009, 10:00 am and October 29th 2009, 04:30 pm and covered six 8-hour shifts (see Table 5). Sevoflurane concentrations are indicated as columns and the numbers of patients in the PACU after sevoflurane anaesthesia are indicated as (….).

Sevoflurane concentrations in the room air of the PACU were measured over a time period of 54 hours from October 27th 2009, 10:00 am until October 29th 2009, 04:30 pm. In total 55 measurements were performed. One measurement was performed per hour followed by a humid blank measurement. The mean sevoflurane concentration over the whole range of time was 27.9 ppbv (±7.2 ppbv; 95% confidence interval). The minimum concentration measured was 8.0 ppbv (October 28th 2009, 06:22 am) and the maximum concentration was 170.2 ppbv (October 27th 2009, 03:10 pm). All room air concentrations in the PACU and the number of patients in PACU after sevoflurane anaesthesia can be seen in Figure 4.

Personnel working in the PACU exclusively are deployed in this area of the hospital and therefore we only calculated the TWA-S values to quantify their exposure to sevoflurane during their 8-hour working shifts. With 45.1 ppbv the highest TWA-S value was calculated for the late (evening) shift between 02:00 pm and 10:00 pm on October 27th 2009. The lowest TWA-S was 8.3 ppbv and was found for the early (morning) shift between 06:00 am and 02:00 pm on October 28th 2009). The TWA-S values for all shifts covered by the MCC-IMS measurements can be found in Table 5.

**Table 5:** **TWA-S values for hospital personnel working in the PACU. The time of measurement included two morning shifts (06:00 am – 02:00 pm), two evening shifts (02:00 pm – 10:00 pm) and two night shifts (10:00 pm – 06:00 am)**

**Date**	**Working time**		**TWA-S [ppbv]**
	**Beginning**	**End**	
October 27th 2009	02:00 pm	10:00 pm	45.1
October 27th 2009	10:00 pm	06:00 am	22.1
October 28th 2009	06:00 am	02:00 pm	8.3
October 28th 2009	02:00 pm	10:00 pm	23.9
October 28th 2009	10:00 pm	06:00 am	18.7
October 29th 2009	06:00 am	02:00 pm	17.1

## Discussion

In our present study we focused on the use of the MCC-IMS technology for the detection and quantification of the volatile anaesthetic agent sevoflurane. Chronic occupational exposure to low concentrations of sevoflurane has been discussed to have adverse effects on the health of health care workers [3]. Even though evidence is not clear on that issue occupational exposure limits and other ceiling value recommendations were published by governmental and health care organizations [4-7]. To answer the question if MCC-IMS could be a fast and reliable alternative to other analytical methods we calibrated the device to sevoflurane concentrations of 10 – 250 ppbv.

We were able to detect sevoflurane in the mode for the detection of negative ions and found a correlation between peak intensity in the MCC-IMS plots and sevoflurane air concentrations. The calibration of the MCC-IMS showed very low LOD and LOQ. Previously, PIS, PTR-MS and PTR-ToF-MS were used for assessing sevoflurane room air concentrations [4,8,9,12,13]. In our study MCC-IMS showed detection limits lower than all these methods – even when compared to PTR-ToF-MS [13]. The LOQ of the MCC-IMS was found to be 0.0189 ppbv and therewith was very low as well. We were not able to find a LOQ for PIS in the literature. Because of very low detection limits MCC-IMS could be a feasible alternative to other methods, especially when low-level contamination with sevoflurane is expected.

To give an example on the performance of MCC-IMS in quantifying sevoflurane, we performed room air measurements during paediatric anaesthesia and in the PACU. In all children general anaesthesia was induced by sevoflurane mask inhalation and was continued as TIVA. We therefore expected the peak concentrations during this procedure and also expected sevoflurane concentrations to decrease in the following measurements. Our results confirmed these expectations. In all 12 patients we found the highest sevoflurane room air concentrations during mask induction with decreasing concentrations over the following measurements up to 70 minutes time of surgery (see Figure 3).

We also monitored the room air of the PACU for 54 hours. As expected our results showed higher concentrations during the evening shifts than during the night shifts or the morning shifts (see Table 5). These findings reflect the number of patients attending the PACU and being discharged from it (see Figure 4). Sevoflurane concentrations in both anaesthesia workplaces clearly did not exceed recommended exposure values [4-7].

Our results partly differ from findings of previous studies. Most authors using PIS for assessing the exposure of hospital personnel to sevoflurane described higher sevoflurane concentrations and TWA-S values. For instance Hoerauf et al. and Schiewe-Langgartner et al. described TWA-S values between 20 ppbv and 2,240 ppbv when sevoflurane was used as the only volatile anaesthetic agent during general anaesthesia [10,11]. On the other hand our results closely match the findings of Rieder et al. and Trefz et al. who assessed sevoflurane concentrations in the PACU using PTR-MS and PTR-ToF-MS. Both authors describe mean room air concentrations of the substance in a low ppbv-range with maxima between 300 and 1,000 ppbv, depending on the number of patients attending the PACU [13,14]. However, room air concentrations monitored with PIS might be overestimated due to the higher detection limits and results should be controlled by using more sensitive technologies, such as MCC-IMS or PTR-(ToF)-MS.

The calibration of the MCC-IMS using a calibration gas generator is a strength of the study. We found a linear correlation for concentrations between 25 and 175 ppbv. However, the upper end of the calibration range did not show a linear correlation anymore, but we expected higher concentrations in the workplace measurements. We decided to use the polynomial correlation to extrapolate the correlation for the higher concentrations, which is a limitation of our study. For the same reason we were not able to describe an upper detection limit of the method, which could be relevant for very high sevoflurane concentrations. However, to our knowledge for none of the other analytical methods an upper detection limit has been described. The assessment of the MCC-IMS’s performance in assessing much higher concentrations of sevoflurane in the room air should be part of future studies. As another limitation we did not use a different analytical method in parallel to the MCC-IMS. Future studies on the performance of PIS, PTR-MS and MCC-IMS should be compared by parallel measurements.

MCC-IMS allowed us to assess very low concentrations of sevoflurane. Compared to PIS the MCC-IMS device used in this study showed a very low LOD and our results showed a significantly lower exposure to sevoflurane than described by authors that used PIS for their measurements at anaesthesia workplaces. The performance of the MCC-IMS device in our study was similar to PTR-MS and PTR-ToF-MS devices used for the detection and quantification of sevoflurane. Both technologies provide very low detection limits and enable assessment of a minimal exposure to volatile anaesthetics. Due to the suitability to miniaturization and the possibility to detect more than one substance at once (e.g. when more than one anaesthetic agent is used in a workplace), MCC-IMS might be a preferable technology for a hospital setting. However, more investigation is needed to assess the performance of MCC-IMS in the quantification of different volatile anaesthetics, the combination of volatile anaesthetics and nitrous oxygen and the combination of several substances.

## Conclusions

Our findings show that MCC-IMS is a reliable alternative method for the quantification of sevoflurane in the room air. MCC-IMS shows significant lower detection limits than comparable methods and has a particular strength in quantifying low-level contaminations of sevoflurane. The results of our room air measurements reflected the logical implications of the study protocol regarding the trends of sevoflurane concentrations in both anaesthesia workplaces. The exposure of the personnel working in these areas did not exceed recommended limitations.
